# Therapeutic drug monitoring vs. pharmacogenetic testing in the context of elevated olanzapine concentrations and prior clozapine intolerability: a case study

**DOI:** 10.1186/s12888-024-06319-5

**Published:** 2024-12-04

**Authors:** Farah Khorassani, Ricardo Azevedo, Reza Farokhpay

**Affiliations:** 1 University of California, Irvine, School of Pharmacy and Pharmaceutical Sciences, 802 W Peltason Dr, Irvine, CA 92617 USA; 2 University of California, Irvine, School of Medicine, 1001 Health Sciences Road, Irvine, CA 92697 USA

**Keywords:** Olanzapine, Clozapine, Therapeutic drug monitoring, Pharmacogenomic testing, Pharmacogenetic testing

## Abstract

**Background:**

Strong evidence for therapeutic drug monitoring exists for olanzapine and clozapine, however, olanzapine therapeutic drug monitoring is often underutilized. Evidence for pharmacogenomic-guided dosing of antipsychotics is not as robust, especially for cytochrome P450 1A2 metabolized agents such as olanzapine and clozapine. Herein, we present a case involving a patient suspected of having poor CYP1A2 metabolism. Therapeutic drug monitoring of olanzapine was employed to guide the titration of clozapine following olanzapine failure. Despite pursuing pharmacogenetic testing, no meaningful results were obtained due to the omission of CYP1A2 variants associated with poor metabolism.

**Case presentation:**

A 32-year-old Caucasian male with schizoaffective disorder-bipolar type, ADHD, and autism spectrum disorder presented with extrapyramidal symptoms due to antipsychotic polypharmacy, resulting in multiple falls. He experienced a partial response to olanzapine 40 mg, thus his dose was increased to 50 mg. Sampling an olanzapine trough revealed a supratherapeutic level of 152 ng/mL. Given his history of EPS and other reported adverse effects from antipsychotics, including clozapine, pharmacogenomic testing was pursued. The patient cross-tapered to clozapine slowly, with the knowledge that the patient would likely exhibit elevated levels of olanzapine. Clozapine was efficacious and tolerated well. As expected, the patient exhibited higher clozapine trough concentrations for someone of his age, ethnicity, and gender. Pharmacogenomic testing yielded no relevant findings relating to olanzapine or clozapine metabolism.

**Conclusion:**

This case highlights the utility of TDM over pharmacogenetic testing for patients on these medications with a suspected alteration in CYP1A2 metabolism. therapeutic drug monitoring emerges as a more practical approach with stronger evidence for its use, particularly in cases of suspected reduced CYP1A2 activity, where suballeles resulting in decreased enzyme function are not readily detectable on standard commercial pharmacogenomic panels.

## Background

Cytochrome P450 subfamily A member 2 (CYP1A2) plays a pivotal role in the metabolism of antipsychotic drugs, particularly olanzapine and clozapine. Olanzapine is primarily metabolized by the liver through the activity of CYP1A2, with only minimal involvement of CYP2D6. Phase II metabolism via UGT1A4 is also involved in olanzapine conjugation [1]. In vivo studies have suggested that olanzapine clearance remains largely unaffected by individuals with reduced CYP2D6 activity [1]. In contrast, clozapine metabolism is more complex, primarily metabolized by CYP1A2, leading to the formation of its major metabolite norclozapine (N-desmethylclozapine) [2]. Minor metabolic pathways through CYP 2C19, CYP2D6 and CYP3A4 also contribute to clozapine metabolism. Notably, individuals characterized as poor metabolizers of CYP2D6 may experience elevated clozapine concentrations due to decreased metabolism per the US Food and Drug Administration product labeling though the evidence of its relevance is questionable in clinical practice [3].

In cases of suboptimal response to antipsychotic treatments, therapeutic drug monitoring (TDM) may be employed to assess the patient’s serum drug concentrations and aid in making informed decisions regarding medication selection. Existing literature strongly supports the implementation of TDM in patients prescribed clozapine (level of recommendation to use TDM = 1), as it suggests enhanced therapeutic efficacy with serum concentrations greater than 350 ng/mL [4]. Olanzapine TDM is also given a level 1 recommendation for TDM, however is not utilized in practice as commonly. Olanzapine TDM may be used in cases of questionable adherence, partial response at maximum doses, or when treatment failure is suspected, with a target range of 20–80 ng/mL [4]. TDM serves an additional purpose by identifying elevated serum concentrations that might otherwise remain unnoticed, particularly in patients with schizophrenia who are unable to effectively communicate adverse effects.

Herein, with written consent from the patient’s guardian, we describe a case of a patient with unexplained elevated olanzapine and a history of prior clozapine intolerability (unknown doses/concentrations). Pharmacogenomic testing was pursued, yielding no pertinent findings.

## Case report

The patient is a 32-year-old conserved, Caucasian male (weight: 83.8 kg, body mass index (BMI) 25.77 kg/m²) with diagnoses of schizoaffective disorder-bipolar type, attention deficit hyperactivity disorder (ADHD), and autism spectrum disorder admitted to an inpatient psychiatric unit from a long-term residential facility after worsening extrapyramidal symptoms (EPS) lead to multiple falls. After a neurological work-up, it was determined that his symptoms were likely secondary to antipsychotic polypharmacy. His medication regimen consisted of ziprasidone 40 mg twice daily, escitalopram 10 mg daily, divalproex sodium extended-release (ER) 1250 mg nightly, perphenazine 16 mg daily and 20 mg nightly, quetiapine 100 mg nightly, and benztropine 1 mg twice daily. The patient had no history of substance use or suicide attempts.

Upon admission to the unit, the patient had a high steppage gait, bilateral upper and lower extremity tremors, upper extremity rigidity, and was dysarthric. The patient had difficulty leaving his bed due to instability, requiring a sitter. The patient was notably responding to internal stimuli, endorsed delusions of persecution, was poorly engaged with interviewers, rarely engaged with peers, and was irritable. Urine toxicology on admission confirmed the presence of benzodiazepines, consistent with his treatment regimen, but was negative for other illicit substances.

Over the course of admission, he was maintained on divalproex sodium ER 1250 mg nightly. Escitalopram was replaced with fluoxetine because the patient’s family felt that it better controlled his irritability. Benztropine was discontinued and trihexyphenidyl was up-titrated to 10 mg twice daily and eventually discontinued as EPS improved. The patient had a history of clozapine use, however, his conservator reported oversedation and other adverse effects, making them hesitant to consent to re-initiate it. In an effort to reduce polypharmacy, perphenazine was discontinued prior to olanzapine initiation. Olanzapine was slowly titrated to 50 mg. The patient exhibited a partial response to olanzapine thus a steady-state, serum olanzapine trough was drawn. The 24-hour trough was found to be 152 ng/mL, (therapeutic range: 20–80 ng/mL). A laboratory sampling error was ruled out after another 12-hour trough was drawn and found to be 173 ng/mL. Per Kelly and colleagues, olanzapine 50 mg administered daily in nonsmoking men should produce, mean concentrations of approximately 127 ng/mL [5]. Given the elevated level, the team completed a comprehensive review to rule out medications or conditions that could slow CYP1A2 metabolism. The patient was receiving divalproex sodium, however, the literature is conflicting as to whether this would increase or decrease concentrations of CYP1A2 metabolized drugs. No definitive CYP1A2 inhibitors were co-administered and there was no caffeine consumption. The patient exhibited no acute weight gain, indications of acute inflammation or medical illness, as evidenced by the absence of systemic symptoms and normal laboratory findings, including C-reactive protein (CRP) levels. The patient was sedated but did not exhibit adverse effects such as xerostomia, orthostasis, or constipation. Olanzapine was lowered and augmented with aripiprazole with no effect. Based on findings from olanzapine TDM in conjunction with the patient’s clinical course, the team obtained consent to initiate clozapine and performed a slow titration given the patient’s history. (See Table 1). Pharmacogenomic testing was obtained at this time to assess for CYP variants that could impact clozapine metabolism and provide information regarding metabolism of other antipsychotics since the patient had a complex history of adverse effects to antipsychotics and often required polypharmacy to control symptoms.

**Table 1:** Medication list and serum concentrations

Admission day (d)	Current Dose (mg)	Concurrent Psychiatric Medications at Initiation	Olanzapine (OLZ) Clozapine (CLZ) and Norclozapine (NOR) Trough Concentrations (ng/ml)
29	Olanzapine 40 mg every night	Trihexyphenidyl 2 mg every morning + 5 mg every night Divalproex sodium ER 1,250 mg at bedtime Fluoxetine 20 mg daily	[OLZ] = 152 (24 h trough)
55	Olanzapine 50 mg every night	Aripiprazole 5 mg daily Trihexyphenidyl5 mg every night Divalproex sodium ER 1,250 mg every night Fluoxetine 10 mg daily	[OLZ] = 173 (12 h trough)
82	Clozapine 37.5 mg	Olanzapine 10 mg every night Aripiprazole 5 mg daily Divalproex sodium ER 1250 mg every night Fluoxetine 10 mg daily Amlodipine 10 mg daily Polyethylene Glycol 17 g daily	[CLZ] = 103/ [NOR] = 26 CLZ Ratio = 3.96
103	Clozapine 75 mg	Aripiprazole 5 mg daily Divalproex sodium ER 1250 mg every night Fluoxetine 10 mg daily Amlodipine 10 mg daily Polyethylene Glycol 17 g daily	[CLZ] = 218 [NOR] = 49 CLZ Ratio = 4.45
117	Clozapine 112.5 mg	Divalproex sodium ER 1250 mg every night Amlodipine 10 mg daily Polyethylene Glycol 17 g daily	[CLZ] = 271 [NOR] = 59 CLZ Ratio = 4.59
133	Clozapine 162.5 mg	Divalproex sodium ER 1250 mg every night Escitalopram 10 mg every night Amlodipine 10 mg daily Polyethylene Glycol 17 g twice daily	[CLZ] = 362 [NOR] = 85 CLZ Ratio = 4.26

**Fig. 1:**
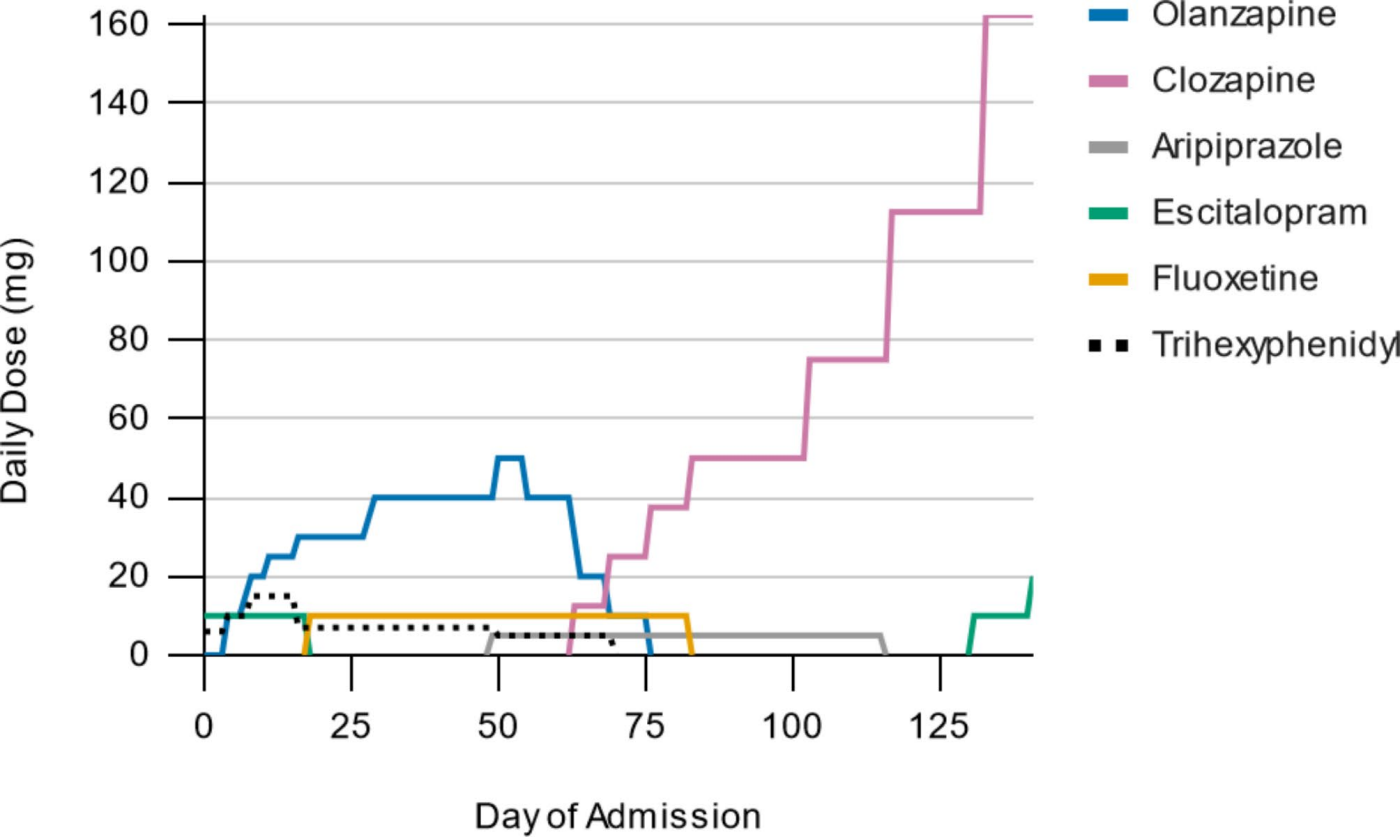
Timeline of medication dosing and titration

The patient’s symptoms responded to clozapine and he was responding to internal stimuli less. He exhibited higher than expected clozapine levels with the first steady-state, 12-hour clozapine trough of 103 ng/mL at a dose of 37.5 mg. The patient also exhibited an elevated clozapine: norclozapine ratio suggesting possible impaired CYP1A2 metabolism. The patient was slowly titrated to a dose of 162.5 mg (serum clozapine 362 ng/mL, norclozapine 85 ng/mL) and maintained. Regarding the patient’s other medications, fluoxetine was discontinued during the titration with no observed impact on serum concentrations, ruling out fluoxetine mediated CYP2D6 inhibition as a contributor to altered antipsychotic metabolism. Escitalopram was eventually restarted because of worsening mood symptoms. See Fig. 1 for medication titrations over the course of the hospitalization.

## Discussion/Conclusions

This case underscores the importance of TDM and its utility over pharmacogenomic testing when aiming to identify altered metabolism in patients receiving olanzapine and clozapine, especially those exhibiting partial response or treatment failure. With the knowledge of the patients supratherapeutic olanzapine level, the treatment team was able to confirm that the patient had a failed trial of olanzapine and better developed a plan to titrate clozapine. The origin of these elevated serum concentrations remains elusive, as the patient was not on any medications inhibiting CYP1A2, and factors such as significant weight change, caffeine consumption, and inflammation measured by CRP, were ruled out. The patient’s reported prior clozapine intolerability was better explained when the olanzapine concentration returned supratherapeutic because both are metabolized by CYP1A2. This provided critical information during the transition to clozapine when deciding how quickly to titrate, monitoring parameters, and the potential target clozapine dose.

Pursuing pharmacogenomic testing was planned to determine if there was a particular etiology related to the altered metabolism and to obtain any guidance on future dose modifications for clozapine or other antipsychotics (see Table 2). Not much could be concluded from the commercial assay utilized as it did not provide information on variants producing reduced olanzapine or clozapine metabolism. The assay did test for the CYP1A2 *1F variant, associated with rapid CYP1A2 metabolism. The patient was found to be a normal UGT1A4 metabolizer, a rapid 2C19 metabolizer, and intermediate CYP2D6 metabolizer, neither of which would alter olanzapine or clozapine metabolism.

**Table 2:** Relevant Patient Genotype and Phenotype Variations

Genotype	Phenotype
CYP1A2, *1F/*1F	Normal metabolizer
CYP2C19, *1/*17	Rapid metabolizer
CYP2D6, *1/*4	Intermediate metabolizer
CYP3A4, *1/*1	Normal metabolizer
UGT1A4, *1/*1	Normal metabolizer

This highlights the importance of carefully selecting pharmacogenomic assays when attempting to gain information on particular variants There are numerous pharmacogenomic assays available that test for different variants, identify patient phenotypes, and provide recommendations for patient management based on results. On assays, variants more commonly identified in the literature as impacting metabolism/outcomes are including on these assays. If looking for a specific variant, it is best to confirm that the assay tests for this to ensure you will be able to obtain relevant results and to confirm the relevance of these variants per available literature. For this patient, CYP1A2 genotyping was the primary CYP of interest, and the *1F was the only suballele tested for. No other 1A2 variants such as *1D or *1K were tested on the assay, which would better reflect reduced CYP1A2 activity. This highlights the importance of delving into specific variants tested for CYPs of interest, especially given the heterogeneity in assays [6]. 

A retrospective study by Czerwensky and colleagues suggested an association between CYP1A2*1D and increased olanzapine concentrations, but limitations, such as a retrospective study design, small sample size, and the presence of environmental factors like coadministration with carbamazepine, valproic acid, and smoking, limit the generalizability of these findings [7].

Regulatory bodies like the Food and Drug Administration (FDA) and the Dutch Pharmacogenetic Working Group (DPWG) do not recommend routine pharmacogenetic testing for clozapine and olanzapine [3, 8]. The 2023 guidelines from the DPWG regarding drug-gene interactions in antipsychotics concluded that there is no observable drug-gene interaction between CYP1A2 and clozapine or olanzapine [8]. This conclusion is backed by studies indicating no significant differences in response or adverse effects among predicted phenotypes and genotype groups.

Lastly, the Clinical Pharmacogenetics Implementation Consortium (CPIC) is in the process of drafting guideline recommendations for pharmacogenetic testing for antipsychotics; recommendations will likely be similar to those of the DPWG. Given the cost and logistical barriers of commercial assays, their use limited in determining CYP1A2 metabolizer status if the patient is a suspected poor metabolizer, though could provide information regarding metabolism of other antipsychotics metabolized by enzymes such as CYP2C19 and CYP2D6.

In conclusion, the case highlights interindividual variability in olanzapine and clozapine metabolism and the potential utility of TDM over pharmacogenetic testing for patients on these medications. TDM emerges as a more practical approach with stronger evidence for its use, particularly in cases of suspected reduced CYP1A2 activity, where suballeles resulting in decreased enzyme function are not readily detectable on standard commercial pharmacogenomic panels. TDM allows for more personalized dosing strategies and is ultimately more likely to provide results that can provide guidance on dosing or medication selection. When considering pharmacogenetic testing for patients on psychotropics, it is important to carefully select the assay and consider the clinical utility of testing based on available evidence-based guidelines.

## Data Availability

No datasets were generated or analysed during the current study.

## Abbreviations

TDM: Therapeutic drug monitoring
ADHD: Attention deficit hyperactivity disorder
CYP: Cytochrome P450
BMI: Body mass index
CRP: C-reactive protein
FDA: Food and Drug Administration
DPWG: Dutch Pharmacogenetic Working Group
CPIC: Clinical Pharmacogenetics Implementation Consortium
